# Longitudinal Changes in Symptoms of Post-Intensive Care Syndrome: A Secondary Analysis of a Scoping Review

**DOI:** 10.31662/jmaj.2025-0040

**Published:** 2025-09-12

**Authors:** Kohei Tanaka, Nobuto Nakanishi, Keibun Liu, Kyohei Miyamoto, Akira Kawauchi, Masatsugu Okamura, Sho Katayama, Kensuke Nakamura

**Affiliations:** 1 Department of Rehabilitation Medicine, Osaka International Medical and Science Center, Osaka, Japan, Karasugatsuji, Tennouji-ku, Osaka, Japan; 2 Division of Disaster and Emergency Medicine, Department of Surgery Related, Kobe University Graduate School of Medicine, Kusunoki-cho, Chuo-ku, Kobe, Japan; 3 Non-Profit Organization ICU Collaboration Network (ICON), Tokyo, Japan; 4 Department of Emergency and Critical Care Medicine, Wakayama Medical University, Kimiidera, Wakayama, Japan; 5 Department of Critical Care and Emergency Medicine, Japanese Red Cross Maebashi Hospital, Asakura-Machi, Maebashi-shi, Gunma, Japan; 6 Berlin Institute of Health Center for Regenerative Therapies (BCRT), Charité - Universitätsmedizin Berlin, Berlin, Germany; 7 Department of Rehabilitation Medicine, Okayama University Hospital, Shikata, Kita-ku, Okayama, Japan; 8 Department of Critical Care Medicine, Yokohama City University Hospital, Fukuura, Kanazawa-ku, Yokohama, Kanagawa, Japan

**Keywords:** post-intensive care syndrome, PICS-F, physical function, cognitive function, mental health, quality of life, long-term outcome

## Abstract

**Background:**

Since the increased interest in post-intensive care syndrome (PICS) and PICS-family, many studies have been conducted. However, the longitudinal changes in PICS symptoms are not clearly understood. This study aimed to summarize the longitudinal symptom changes in each PICS domain, including physical, cognitive, mental health, quality of life, and family.

**Methods:**

In this secondary analysis study, we identified the studies that conducted longitudinal PICS assessments published between 2014 and 2022. The most frequently used assessment tools in each domain were defined as representative methods, and results of included studies were summarized by each PICS domain. The collected data were grouped by the following: within 3 months (baseline), at 3 months, 6 months, and annually thereafter. The Wilcoxon rank-sum test was used to compare changes from baseline.

**Results:**

Of the 5,160 studies screened, 76 studies were selected in this study. The percentage predicted value of the 6-minute walk test as an indicator of physical function significantly improved from baseline to 6 months (median [interquartile range], from 45.9 [32.0-63.0] to 65.0 [57.8-72.8], p = 0.04), and continued to improve during the 5-year follow-up period. Montreal Cognitive Assessment for cognitive assessment and Impact of Event Scale-Revised for mental health assessment did not show statistically significant change. The anxiety score of the Hospital Anxiety and Depression Scale (HADS) improved from baseline to the 12-month follow-up (from 6.8 [4.0-8.5] to 4.3 [3.3-5.3], p = 0.04). The anxiety and depression scores of the HADS in family members did not show statistically significant change. The physical component summary of the Short Form-36 for quality of life assessment increased from baseline to the 12-month follow-up (from 31.3 [25.5-37.1] to 40.6 [36.9-50.0], p = 0.03); however, the mental component summary of the Short Form-36 was not changed with statistical significance.

**Conclusions:**

Although the physical and mental domains showed significant longitudinal improvements, PICS symptoms were long-lasting in all domains with varying severity.

## Introduction

Intensive care medicine has significantly improved, and the mortality rate in critically ill patients has decreased ^(1), (2)^. Post-intensive care syndrome (PICS) is a long-lasting physical, cognitive, and mental health disorder that occurs during or after intensive care. Family members of patients with intensive care unit (ICU) treatment may experience psychological complications such as anxiety, depression, and acute stress disorder, which are known as PICS-family (PICS-F). Owing to the growing interest in PICS, several studies have been conducted. Approximately 60% of ICU survivors reportedly experienced new physical, mental, and/or cognitive disorders at 1 year following ICU admission ^(3)^. Generally, ICU survivors exhibit some improvement in physical dysfunction, cognitive dysfunction, and mental health disorders ^(4), (5)^. Especially in mental health status, its symptoms sometimes change with remission and/or recurrence ^(6)^. Hereinabove, several studies have revealed the prevalence of post-ICU impairments and reported the chronological changes in their symptoms. However, the chronological changes in PICS symptoms have not been systematically analyzed. PICS studies are highly heterogeneous owing to the disease categories, severity, comorbidity, and various confounding factors in each study. Various methods have been employed for PICS assessment, and several different assessment tools have been used to report on each study. Consequently, statistically integrating the research data is challenging. Therefore, this study aimed to conduct an exploratory scoping review and summarize the long-term longitudinal changes in PICS symptoms.

## Materials and Methods

### Study design

In this secondary analysis, we used the data from a scoping review that identified PICS follow-up studies and conducted a modified Delphi meeting to recommend the assessment instruments for PICS ^(7)^. The original study was registered as a clinical trial (University Hospital Medical Information Network (UMIN) Clinical Trials Registry: 000049634). As this is a secondary analysis study of the scoping review, approval from an ethics committee was not obtained.

### Studies from the original scoping review

The original scoping review screened studies that focused on PICS outcomes of patients with critical illness, including the physical, cognitive, mental health, quality of life (QOL), family domains, and others (e.g., pain and sleep), from January 2014 to December 2022 from the following databases: Cochrane Central Register of Controlled Trials in the Cochrane Library, Medical Literature Analysis and Retrieval System Online via PubMed, and Cumulative Index to Nursing and Allied Health Literature. The search strategies are shown in Supplemental File 1. The following were the inclusion criteria: adult ICU survivors (≥18 years old) and their family members, observational study and randomized controlled trial, and assessment of any PICS outcome at hospital discharge or thereafter. The following were the exclusion criteria: studies with study designs, including reviews, protocols, trial registries, case reports, and conference abstracts; and studies in languages other than English. The first screening extracted 5,160 studies, and 754 studies were included after the second screening in the original study (Supplemental File 2).

### Study screening in this scoping review

Overall, 754 articles were screened to identify which PICS assessment tools were used and when the assessment was conducted. Studies with only a single-time assessment were excluded to observe longitudinal changes. PICS assessment tools were categorized by PICS domain (physical, cognitive, mental health, QOL, and family). Assessment timings had several variations, such as 1 month following hospital discharge, 1 month following ICU discharge, and 1 month follow-up. ICU and hospital discharge were summarized into “discharge,” and the timing of the assessments was calculated on the basis of the period from discharge. Assessment timings were categorized by months from discharge. The data were rounded in cases where the timings were described in days or with fractions.

### Longitudinal PICS assessments

As various assessment tools were employed in the included studies, representative assessment tools in each PICS domain were selected. We identified the assessment tools used in each study included in this review one by one and counted how many times each assessment tool was used in all studies. Thereafter, the assessment tool that was most frequently used in each domain was selected as the representative data. The results of the studies are summarized in the line graphs for each PICS domain. In cases where the results for the intervention and control groups were stated, the data from the control group were selected. For studies without detailed data, the figures were analyzed using automeris.io (https://automeris.io/index.html).

### Other variables

The study type was categorized into randomized controlled trial, interventional study, and observational study. The major reasons for ICU admission and the ICU type (e.g., medical and surgical) were summarized as representative of the most common in each study. Furthermore, data on the sample size, age, disease severity (sequential organ failure assessment score and acute physiology and chronic health evaluation II score), length of ICU stay, and length of hospital stay were collected. In cases where the results for the intervention and control groups were stated, the data from the control group were selected.

### Statistical analysis

Data were presented as medians (first-third quartiles). The collected data were grouped by the following: <3 months (baseline), at 3 months, 6 months, and annually thereafter to analyze the trends in chronological changes. Mixed-effects models for repeated measures (MMRM) were used to analyze the longitudinal changes in PICS symptoms. The longitudinal changes were assessed in comparison with baseline values. Sensitivity analyses were also conducted with MMRM. The first model, for considering coronavirus disease 2019 (COVID-19), whether the patients with COVID-19 were included or not, was included as a fixed effect. The second model, for adjusting disease severity and background disease, length of ICU stay, and background disease, was included as fixed effects. Background diseases were categorized by COVID-19, sepsis, respiratory disease, surgical, medical, and other. A p-value of <0.05 was considered statistically significant. All statistical analyses were conducted using JMP statistical software version 13.1.0 (SAS Institute Inc., Cary, NC, USA).

## Results

**Figure 1.**
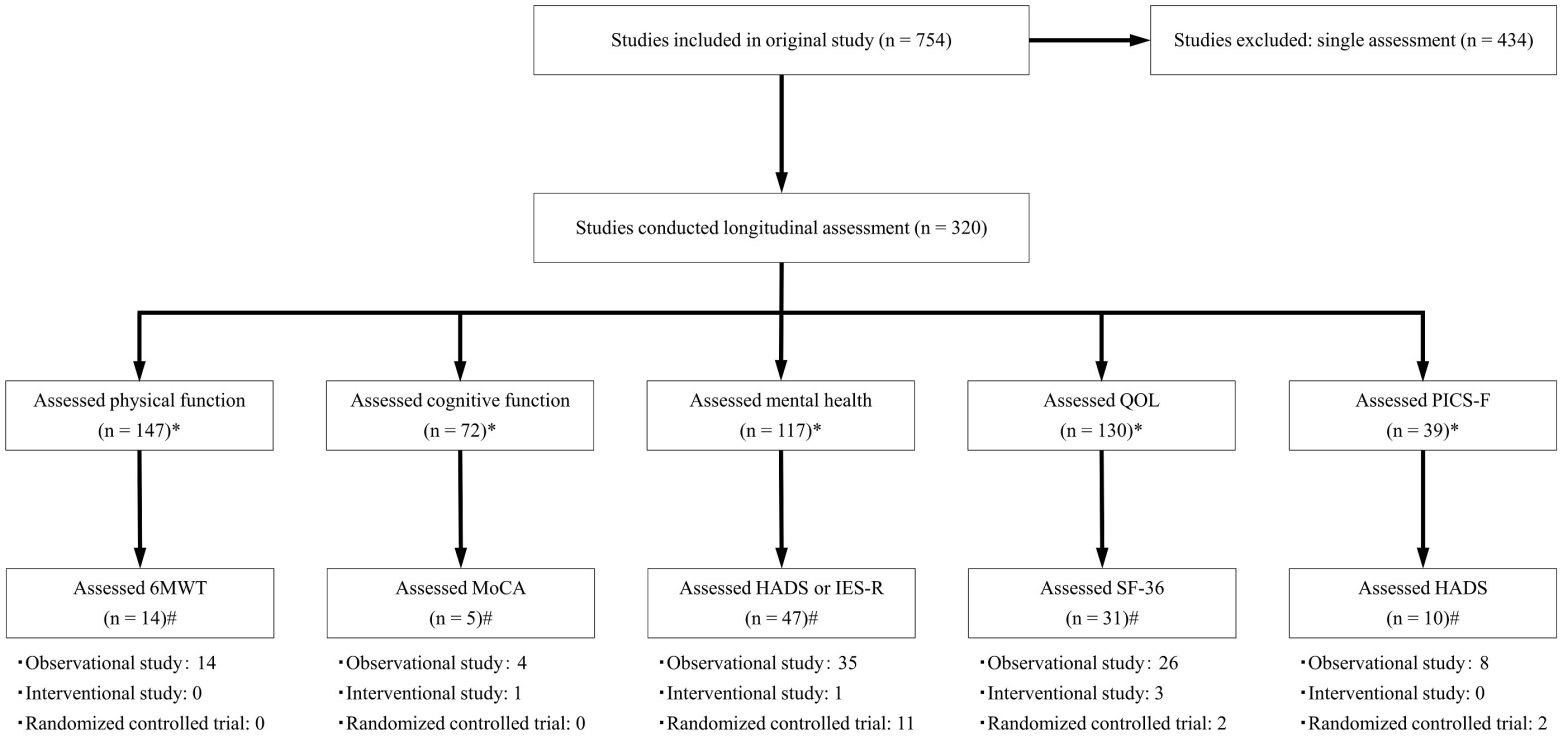
Article selection process. *, #: When several assessments are conducted in the same study, the number of studies is counted for each assessment tool.

The article selection process is shown in Figure 1. The longitudinal PICS assessments were conducted in 320 studies. The following tools were selected: 6-minute walk test (6MWT) for physical impairment, Montreal Cognitive Assessment (MoCA) for cognitive impairment, Hospital Anxiety and Depression Scale (HADS) for anxiety and depression, Impact of Event Scale-Revised (IES-R) for post-traumatic stress disorder (PTSD), Short Form-36 (SF-36) for QOL, and HADS for anxiety and depression for PICS-F. A total of 76 studies (observational studies, 60; randomized controlled trials, 11; and interventional studies, 5) conducted the selected assessments. The characteristics of these 76 studies are shown in Table 1 and listed in Supplemental File 3. The major reasons for ICU admission were COVID-19, sepsis, acute respiratory distress syndrome, and postoperative care in 13, 12, 8, and eight studies, respectively. Overall, 49 studies had fewer than 100 participants, and the median age of the participants was 56.0 (52.0-62.3) years.

**Table 1.** Characteristics of the reviewed studies.

Characteristics	Total	Physical	Cognitive	Mental	QOL	PICS-F
	(n = 76)	(n = 14)	(n = 5)	(n = 47)	(n = 31)	(n = 10)
Study type						
Observational study	60 (78.9)	14 (100)	4 (80.0)	35 (74.5)	26 (83.8)	8 (80.0)
Interventional study	11 (14.5)	0 (0)	1 (20.0)	1 (2.1)	3 (8.1)	0 (0)
Randomized controlled trial	5 (6.6)	0 (0)	0 (0)	11 (23.4)	2 (5.4)	2 (20.0)
Major reasons for ICU admission
COVID-19	13 (17.1)	2 (14.3)	2 (40.0)	10 (21.3)	3 (9.7)	
Sepsis	12 (15.8)	1 (0.7)	1 (20.0)	8 (17.0)	5 (16.1)	
ARDS	8 (10.5)	6 (42.9)	1 (20.0)	6 (12.8)	5 (16.1)	
Surgery	8 (10.5)	1 (0.7)	1 (20.0)	3 (6.4)	2 (6.5)	
Cardiovascular disease	4 (5.3)	0 (0)	0 (0)	1 (2.1)	3 (9.7)	
Other	6 (7.9)	2 (14.3)	0 (0)	3 (6.4)	4 (12.9)	
Not described	17 (22.4)	2 (14.3)	0 (0)	16 (34.0)	9 (29.0)	
Family members	8 (10.5)					10 (100)
ICU type						
Medical	45 (59.2)	12 (85.7)	4 (80.0)	29 (61.7)	25 (80.6)	
Surgical	9 (11.8)	1 (0.7)	1 (20.0)	5 (10.6)	2 (6.5)	
Stroke	3 (3.9)	0 (0)	0 (0)	3 (6.4)	0 (0)	
Not described	11 (11.5)	1 (0.7)	0 (0)	9 (19.1)	4 (12.9)	
Family members	8 (10.5)					10 (100)

ARDS: acute respiratory distress syndrome; COVID-19: coronavirus disease 2019; ICU: intensive care unit.

**Figure 2.**
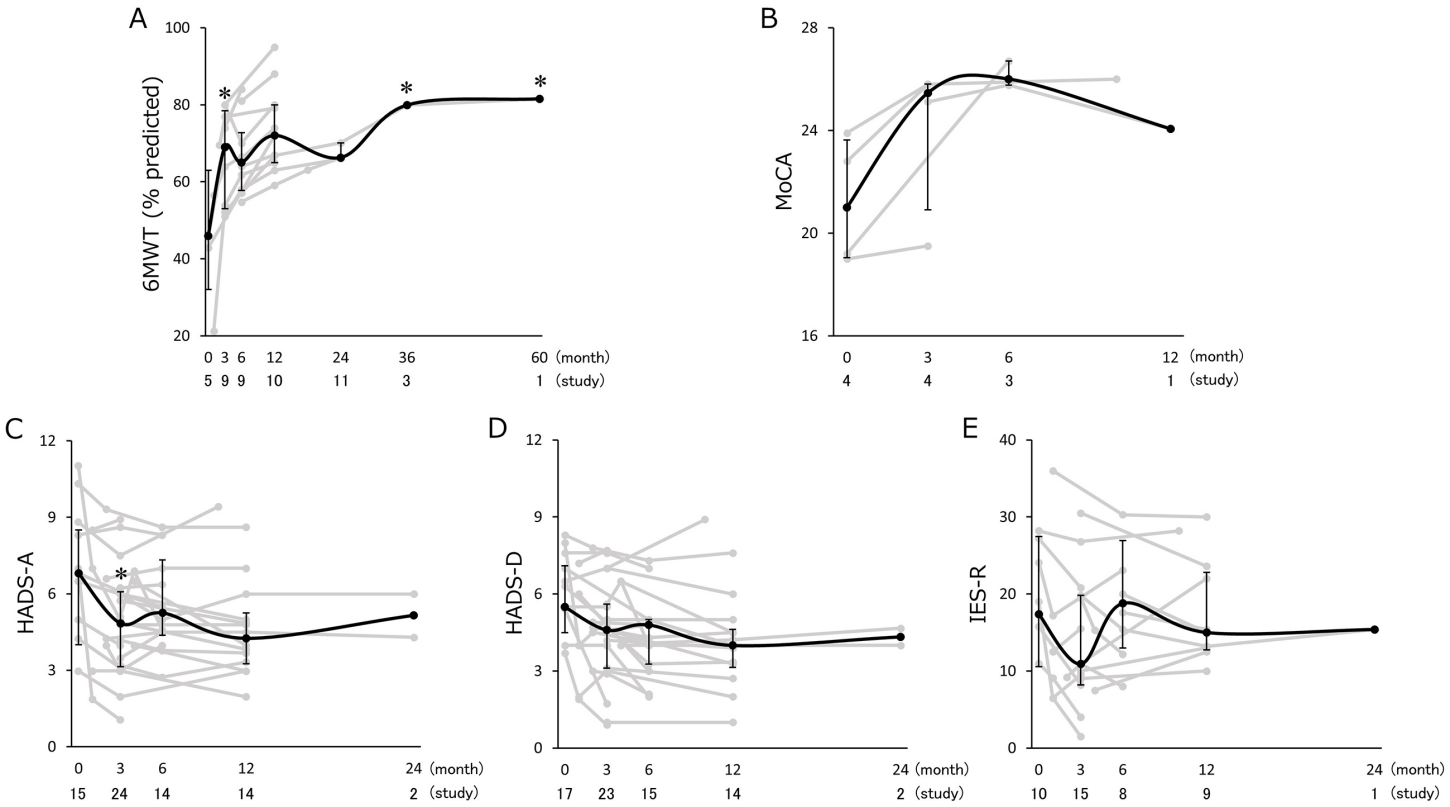
Longitudinal changes in physical function, cognitive function, and mental health. Summary data of the (A) percentage predicted value of the 6-min walk test, (B) Montreal Cognitive Assessment, Hospital Anxiety and Depression Scale (HADS) for (C) anxiety and (D) depression, and (E) Impact of Event Scale-Revised. Data for each study are shown as gray lines, with the medians (interquartile ranges) shown as black lines. The number of included studies and the sum of the sample size are listed under the representative assessment period. Asterisks indicate statistically significant differences from baseline.

Fourteen studies conducted longitudinal assessment for the 6MWT and five studies for the MoCA. The HADS-anxiety-and-depression were longitudinally assessed in 29 and 30 studies, respectively. The IES-R was assessed in 19 studies repeatedly. The longitudinal changes in physical function, cognitive function, and mental health are shown in Figure 2. The results of the 6MWT were reported as percentage predicted value compared with healthy participants more frequently than distance. The calculation of the percentage predicted value of the 6MWT was based on established norms ^(8)^. The 6MWT scores significantly increased from 45.9 (32.0-63.0) at baseline to 69.0 (53.0-78.5) at 3 months (p < 0.01), 65.0 (57.8-72.8) at 6 months (p = 0.04), 79.9 at 3 years (p < 0.01), and 81.5 at 5 years (p < 0.01). The results of sensitivity analyses showed significant change at 3 months (first model: p = 0.04, second model: p < 0.01), 3 years (first model: p = 0.04, second model: p < 0.01), and 5 years (first model: p = 0.03, second model: p < 0.01); however, they did not show statistically significant change at 6 months (first model: p = 0.15, second model: p = 0.28). The MoCA and HADS-depression did not show statistically significant changes. The HADS-anxiety was significantly decreased from 6.8 (4.0-8.5) at baseline to 4.3 (3.3-5.3) at 12 months (p = 0.04). The result of the sensitivity analysis with the second model, however, was not consistent with this result. Sensitivity analysis showed a significant decrease at 3 months with a score of 4.8 (3.1-6.1) (p = 0.03). The IES-R score did not show significant change without adjusting confound factors; however, the sensitivity analysis with the second model showed a significant decrease from 17.4 (10.6-27.5) at baseline to 11.0 (8.2-20.1) at 3 months (p = 0.04).

**Figure 3.**
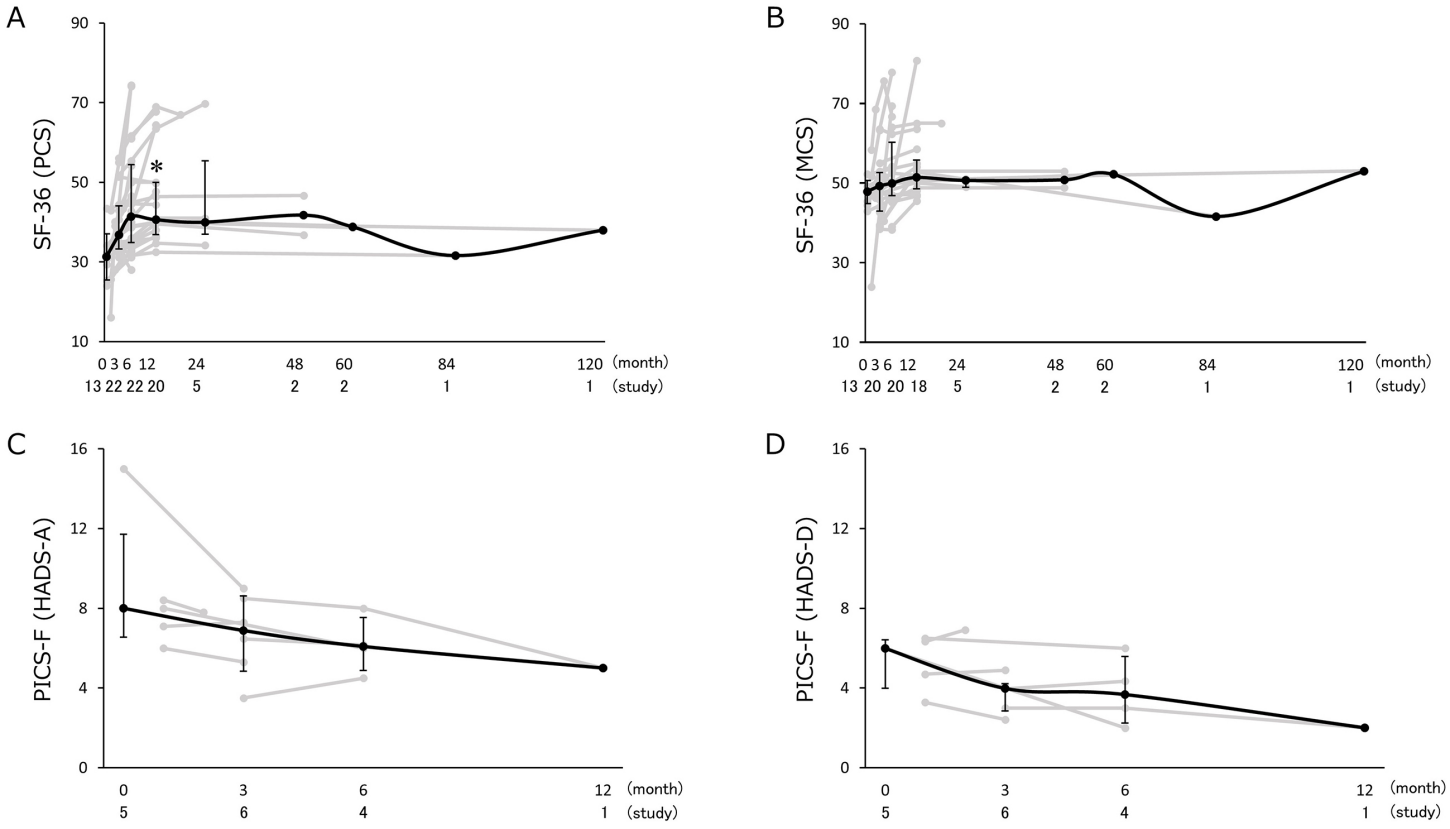
Longitudinal changes in quality of life and PICS-F scores. Summary data of the (A) physical component summary (PCS) and (B) mental component summary (MCS) of the Short Form-36 (SF-36). Summary data of the HADS for (C) anxiety and (D) depression of family members. Data for each study are shown as gray lines, with the medians (interquartile ranges) shown as black lines. The number of included studies and the sum of the sample size are listed under the representative assessment period. Asterisks indicate statistically significant differences from baseline. HADS: Hospital Anxiety and Depression Scale; PICS-F, post-intensive care syndrome family.

The physical component summary (PCS) of the SF-36 was longitudinally assessed in 30 studies, and the mental component summary (MCS) of the SF-36 was assessed in 28 studies. The HADS for family members was assessed in 10 studies. The results of the longitudinal changes in QOL and PICS-F represented in the HADS are shown in Figure 3. The PCS of the SF-36 at 12 months (40.6 [36.9-50.0]) was significantly increased from baseline (31.3 [25.5-37.1]) (p = 0.03). Sensitivity analysis with the first model also showed the same result (p = 0.04), and the second model showed the same trend without statistical significance (p = 0.06). The MCS of the SF-36 did not show a statistically significant change. The longitudinal changes in the PICS-F assessed using the HADS were not statistically significant.

## Discussion

This scoping review is the first study to summarize the longitudinal changes in PICS symptoms by each domain. The physical function continuously and significantly improved during the long-term following period. The physical component of the QOL score and anxiety significantly improved at 1 year following discharge; however, the long-term changes in cognitive function, depression, PTSD, mental component of the QOL score, and PICS-F were not significantly changed. Our findings provide valuable insights into the general trends of chronological changes in PICS symptoms.

The results of the 6MWT, as a physical function indicator, showed longitudinal improvement. In the study showing the greatest improvement, the 6MWT improved from 21% to 65% during the 1-year follow-up ^(9)^. The minimal important difference in the 6MWT in survivors of acute respiratory failure was 3%-5% ^(10)^. Almost all of the reviewed studies reported improvements that exceeded this minimal important difference at every assessment period. Nevertheless, patients following critical care experienced persistent long-term physical impairments. The primary causes of physical impairment following intensive care are muscle atrophy, muscle weakness, and pulmonary dysfunction ^(11)^. Most patients develop severe weight loss on ICU discharge, and increasing lean body mass is difficult; however, total body mass and fat mass may increase ^(12)^. Partial denervation of muscles due to ICU-acquired weakness is largely unchanged and is observed in >90% of patients with prolonged ICU stay up to 5 years following discharge ^(13)^. This event could cause prolonged muscle weakness. Within 6-12 months following critical care, pulmonary function recovers to almost normal, except for a persistent decline in the carbon monoxide diffusion capacity ^(11), (14), (15)^. These long-lasting factors may explain the prolonged physical impairment following critical care.

For cognitive impairment, the MoCA was the most frequently used assessment tool; however, in this review, only five studies that used the MoCA were included. Nersesjan et al. reported a remarkable improvement in the MoCA among patients following COVID-19 infection during the 6-month follow-up ^(16)^. However, the other four studies showed only slight changes in the MoCA. Considering the results of this scoping review, cognitive impairment that develops following intensive care may persist longitudinally. The biological mechanism of long-term cognitive impairment remains unclear ^(17)^. However, several risk factors for cognitive impairment, including delirium and sleep disturbance in the ICU, have been identified ^(18)^. Exercise therapy has the beneficial effects of protecting and recovering cognitive function by reducing the risk of delirium and maintaining circadian rhythms ^(19)^. Moreover, improved physical function by exercise therapy may facilitate cognitive improvement by increasing social participation. Future research should clarify the effects of exercise therapy on long-term cognitive function by reducing the risk of cognitive dysfunction and enhancing social participation by improving physical function.

In this review, mental health disorders are represented by the HADS and IES-R. Some studies have reported significant improvement during the short-term follow-up; however, most studies have demonstrated slight changes during the long-term assessments. Symptoms of anxiety, depression, and PTSD persisted once developed and may occasionally show remission and/or recurrence ^(6)^. This review noted a similar trend of challenges in symptom amelioration. Although the complex and multifactorial etiology of mental health disorders has been revealed ^(20), (21), (22)^, the pathophysiology of mental health disorders following critical care remains unclear. Nevertheless, physical function has been reported as a robust predictor of improving mental health disorders ^(6)^. For example, hand grip strength, a major physical function indicator, has been reported to be negatively correlated with the HADS ^(23)^. Moreover, exercise therapy positively affects mental health by improving physical function and facilitating the cultivation of behavioral mechanisms of change ^(24)^. Continuous intervention by a comprehensive multidisciplinary team, including staff who can commit to recovering physical function, may be essential to improve mental health disorders following intensive care.

The SF-36 comprises eight health concept domains and provides summary scores for the physical and mental components. In both components, some studies did not show major changes for several years; however, some studies showed improvements during the first year of the follow-up period. Especially at 1 year following discharge, the physical component appears more likely to improve than the mental component. A previous study has revealed that the SF-36 score for the physical component improved in parallel with the improvement in the 6MWT ^(11)^. As demonstrated in this review, the significant improvement in 6MWT would be shown within several months after hospital discharge. Furthermore, mental health disorders, including anxiety, depression, and PTSD, were frequently persistent for a long time ^(6)^. The characteristics of the physical and mental health impairments may explain the differences in longitudinal changes between the QOL components.

Regarding the PICS-F, some studies have reported short-term improvements in HADS scores; however, the general trend was not significantly changed in the long term. The prevalence of mental health symptoms improved within the short term and remained stable thereafter ^(25)^. This tendency was also observed in this review. The PICS-F can be categorized into psychological and non-psychological factors. Psychological stress is caused by various factors, such as a loved one having a serious illness, a difficult experience with death, and lifestyle changes for care. Effective coping mechanisms and support from significant others can alleviate these stressors. Non-psychological factors include socioeconomic challenges and physical symptoms. Previous studies have revealed that family members showed significant lifestyle disruption, employment reduction, and financial depletion, which persisted for 1 year ^(26), (27)^. Families frequently experience fatigue and poor sleep quality, which have been prolonged for several months and are associated with psychological symptoms ^(28), (29)^. As shown in this review, PICS symptoms persist long term; therefore, PICS-F symptoms would also be long-lasting.

This study had some limitations. The major limitation of this review lies in the heterogeneity of patient backgrounds across included studies, precluding direct comparison of results. We conducted sensitivity analyses to reduce the influence of differences in patient background. However, due to the heterogeneity of the included studies, the influence of patient background would not be completely excluded. Especially in MoCA, the statistical analysis adjusted for the presence of COVID-19 could not be conducted due to the small number of studies. Furthermore, some assessment time points with a small number of included studies may have a negative influence on the statistical results. The significance of this study lies in the exploratory review of previous studies, with the results of each included study being summarized to elucidate general trends in longitudinal symptom change. To analyze the data on the basis of disease category and severity, accumulating more evidence with standardized assessment tools and assessment timing would be necessary. Second, this review included several studies on COVID-19 as studies on PICS. However, long COVID has been reported to be different from PICS ^(30), (31)^. If a clear distinction is made between PICS and long COVID, the results of this study should be interpreted with caution. Furthermore, several studies were excluded because we selected the most frequently used assessment tools as representative indicators, which is also a limitation of this study. Recommendations on assessment tools have been recently published ^(7), (32)^; however, it appears that various assessment tools are still employed in studies. It is desirable for studies on PICS to use standardized assessment tools to facilitate easier comparison of the results of different studies. Third, the timing of the PICS assessments could not be adjusted in this review. As the follow-up periods were counted from ICU discharge, hospital discharge, or other timepoints, they were summarized into “discharge.” An inaccuracy exists depending on the length of ICU or hospital stay. We collected data on the length of hospital and ICU stay for all patients in each study (Supplemental File 3); however, we were unable to match the timing of the assessments as several studies did not list these data. Furthermore, the grouping of assessment timings was broadly defined, and there is a potential implication of oversimplifying the actual recovery trajectories. In a previous study that reviewed the temporal trends in QOL after cardiac surgery, the assessment timings were categorized broadly, similar to our study ^(33)^. Due to the variety of assessment timings among previous studies, we had to define the grouping broadly. Therefore, the results of this review should be considered as information to capture the major trends in longitudinal PICS symptoms. Lastly, the severity of the functional impairment was not carefully considered. The cutoff score of the MoCA for predicting mild dementia was 25 points ^(34)^. Scores of <8 and 25 points of the HADS and IES-R, respectively, suggest the presence of symptoms ^(35), (36)^. Several studies in this review had scores lower than the cutoff values. For example, Capin JJ reported the MoCA scores of 19.0 and 19.5 at baseline and 3 months follow-up ^(37)^. Further, Heins HE showed that the HADS score was less than six points and the IES-R score was less than 20 points during the 2-year follow-up period ^(5)^. Therefore, the slight longitudinal changes observed in some studies could be attributed to the mild disability.

The physical function and QOL score for the physical component following intensive care may improve, particularly at 1 year following discharge. The QOL score for mental health and depressive symptoms gradually improved; however, cognitive function, anxiety, PTSD, and PICS-F persisted in the long term. Although significant improvements were observed, complete recovery of impaired functions, even in the long term, appears to be challenging in all areas of PICS.

## Article Information

### Author Contributions

Kohei Tanaka: Conceptualization, methodology, investigation, writing - original draft.

Nobuto Nakanishi: Conceptualization, methodology, project administration, writing - review and editing.

Keibun Liu, Kyohei Miyamoto, Akira Kawauchi, Masatsugu Okamura, Sho Katayama, and Kensuke Nakamura: Conceptualization, methodology, supervision, writing - review and editing.

### Conflicts of Interest

None

### IRB Approval Code and Name of the Institution

The original study was registered as a clinical trial (UMIN Clinical Trials Registry: 000049634). As this is a secondary analysis study of the scoping review, approval from the Institutional Review Board was not obtained.

## Supplement

Supplementary Material
