# Supplementary material for: Longitudinal Changes in Symptoms of Post-Intensive Care Syndrome: A Secondary Analysis of a Scoping Review
Source: JMA J. 2025 Sep 12;8(4):1089–97. doi: 10.31662/jmaj.2025-0040 (PMC12598277; doi:10.31662/jmaj.2025-0040)
Supplement: Supplementary Material [file 2433-3298-8-4-1089-s001.pdf]

Search strategy

Databases

- MEDLINE
- CENTRAL
- CINAHL

Among 6972 identified records, 5160 were included after the deletion of duplicates.

MEDLINE (via PubMed) search strategy (Searched in AM–08:10, November 27, 2022)

	Search formula	Results
#1	(intensive care[tiab] OR ICU[tiab] OR ICUs[tiab] OR "intensive care units"[MeSH Terms] OR intensive therapy[tiab] OR critical care[tiab] OR "critical care"[MeSH Terms]) AND (survivors[tiab] OR postintensive care syndrome[tiab] OR post intensive care syndrome[tiab] OR PICS[tiab] OR family[tiab]) AND (impairment[tiab] OR physical[tiab] OR cognitive[tiab] OR mental[tiab] OR posttraumatic stress disorder[tiab] OR depression[tiab] OR anxiety[tiab] OR “activities of daily living"[MeSH Terms] OR ADL[tiab] OR quality of life[tiab] OR "quality of life"[MeSH Terms] OR morbidity[tiab] OR "morbidity"[MeSH Terms]) NOT (animals[mh] NOT humans[mh])	5835
#2	Limits: 2014/Jan/1 – present	3471

Cochrane Central Register of Controlled Trials search strategy (Searched in A–08:46, November 27, 2022)

#1	("intensive care"):ti,ab,kw	27771
#2	("ICU"):ti,ab,kw	16086
#3	("ICUs"):ti,ab,kw	1744
#4	MeSH descriptor: [Intensive Care Units] explode all trees	4145

#5	("intensive therapy"):ti,ab,kw	1061
#6	("critical care"):ti,ab,kw	4519
#7	MeSH descriptor: [Critical Care] explode all trees	2238
#8	#1 OR #2 OR #3 OR #4 OR #5 OR #6 OR #7	37647
#9	("survivors"):ti,ab,kw	12622
#10	("postintensive care syndrome"):ti,ab,kw	54
#11	("post intensive care syndrome*"):ti,ab,kw	70
#12	("PICS"):ti,ab,kw	117
#13	("family"):ti,ab,kw	39219
#14	#9 OR #10 OR #11 OR #12 OR #13	51355
#15	("impairment"):ti,ab,kw	38293
#16	("physical"):ti,ab,kw	142628
#17	("cognitive"):ti,ab,kw	84978
#18	("mental"):ti,ab,kw	70589
#19	("posttraumatic stress disorder"):ti,ab,kw	5517
#20	(“depression”):ti,ab,kw	90609
#21	("anxiety"):ti,ab,kw	64582
#22	MeSH descriptor: [Activities of Daily Living] explode all trees	10374
#23	("ADL"):ti,ab,kw	4159
#24	("quality of life"):ti,ab,kw	135560
#25	MeSH descriptor: [Quality of Life] explode all trees	29546
#26	("morbidity"):ti,ab,kw	43154
#27	MeSH descriptor: [Morbidity] explode all trees	15984
#28	#15 OR #16 OR #17 OR #18 OR #19 OR #20 OR #21 OR #22 OR #23 OR #24 OR #25 OR #26 OR #27	471247

#32	#8 AND #14 AND #28	1112
#33	Limits: trial	1087
#34	Limits: 2014/Jan/1 – present	823

CINAHL (Searched in AM–9:17, November 27, 2022)

#1	("intensive care" OR "ICU" OR "ICUs" OR MH "intensive care units" OR "intensive therapy" OR "critical care" OR MH "critical care") AND ("survivors" OR "postintensive care syndrome" OR "post intensive care syndrome" OR "PICS" OR "family") AND ("impairment" OR "physical" OR "cognitive" OR "mental" OR "posttraumatic stress disorder" OR "depression" OR "anxiety" OR MH "activities of daily living" OR "ADL" OR "quality of life" OR MH "quality of life" OR "morbidity" OR MH "morbidity")	4315
#2	Limits: 2014/Jan/1 – present	2678

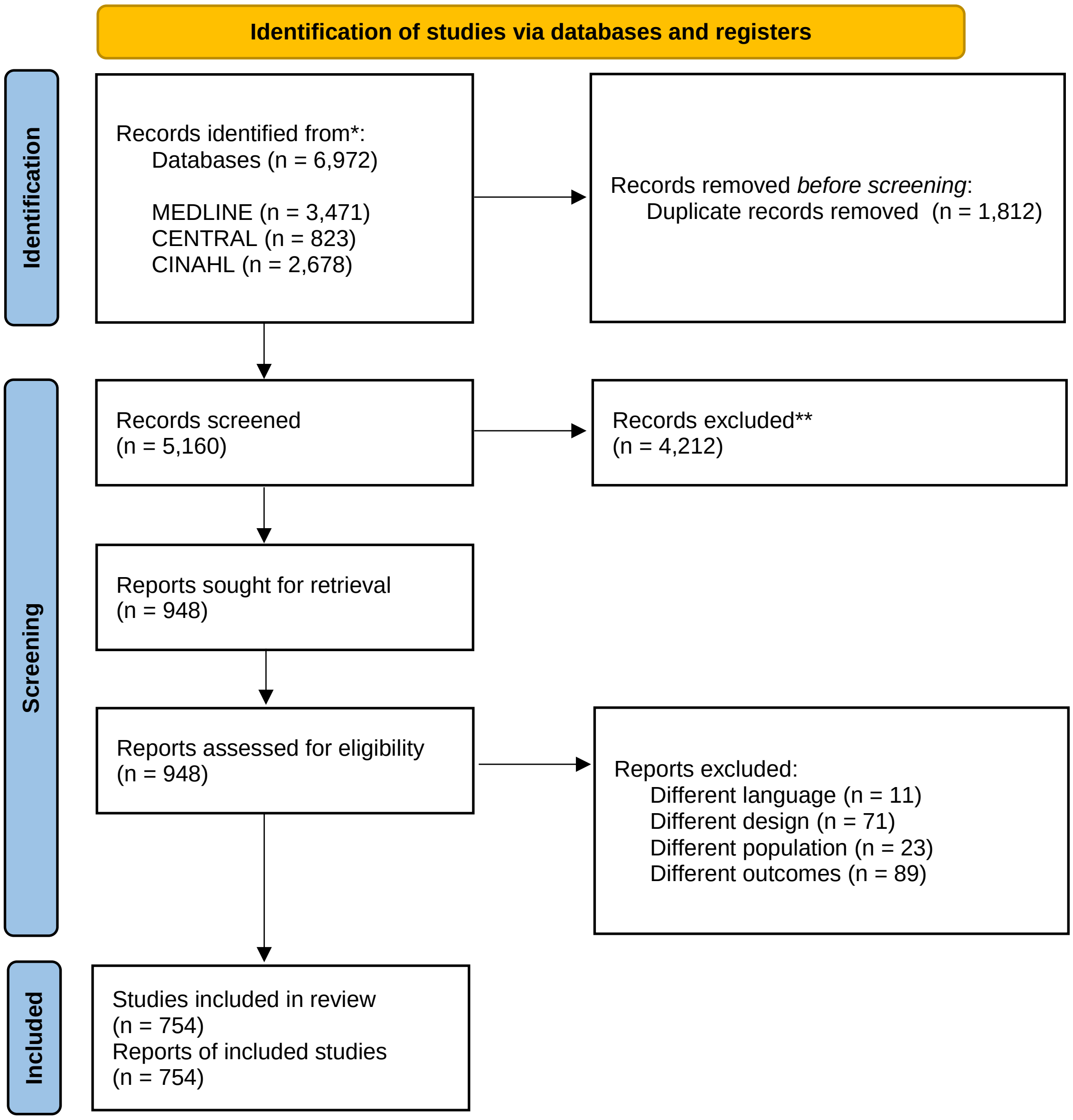
Identification of studies via databases and registers
Identification
Records identified from*:
Databases (n = 6,972)
MEDLINE (n = 3,471)
CENTRAL (n = 823)
CINAHL (n = 2,678)
Records removed *before screening*:
Duplicate records removed (n = 1,812)
Screening
Records screened
(n = 5,160)
Records excluded**
(n = 4,212)
Reports sought for retrieval
(n = 948)
Reports assessed for eligibility
(n = 948)
Reports excluded:
Different language (n = 11)
Different design (n = 71)
Different population (n = 23)
Different outcomes (n = 89)
Included
Studies included in review
(n = 754)
Reports of included studies
(n = 754)

No	Domain	Title	Study type	Sample size	Age	Major reasons for ICU admission	SOFA score	APACHE II score	Starting point for counting the assessment period	Length of ICU stay	Length of hospital stay
1	6MWT	Physical complications in acute lung injury survivors: a two-year longitudinal prospective study	Observation	222	49 (40-58)	Pneumonia	11 (8-15)	26 (20-33)	ICU discharge	13 (7-21)	
2	6MWT	Factors associated with missed assessments in a 2-year longitudinal study of acute respiratory distress syndrome survivors	Observation	196	49 (40-58)	ARDS			Hospital discharge		
3	6MWT	Physical declines occurring after hospital discharge in ARDS survivors: a 5-year longitudinal study	Observation	193	49 (41–58)	ARDS	9 (7–11)	23 (19–28)	Hospital discharge	15 (10–23)	
4	6MWT	Association Between Tracheostomy and Functional, Neuropsychological, and Healthcare Utilization Outcomes in the RECOVER Cohort	Observation	224	58.6 (±15.4)	Not mentioned		22.5 ± 7.8	ICU discharge	16 (12-22)	31 (22-46)
5	6MWT	Life after COVID-19: the road from intensive care back to living - a prospective cohort study	Observation	67	62 (57–68)	COVID			ICU discharge	20 (12-33)	
6	6MWT	An observational study investigating the use of patient-owned technology to quantify physical activity in survivors of critical illness	Observation	39	56 (44-65)	Not mentioned			ICU discharge	6 (4-10)	18 (12-28)
7	6MWT	Physical activity, muscle strength, and exercise capacity 3 months after severe sepsis and septic shock	Observation	72	53.4 (±17.6)	Sepsis	9.0 (6–12)	20.0 ± 6.6	Hospital discharge	10.4 ± 11.1	
8	6MWT	Physical, cognitive and mental health outcomes in 1-year survivors of COVID-19-associated ARDS	Observation	98	Not mentioned	ARDS			ICU discharge		
9	6MWT	High occurrence of postintensive care syndrome identified in surgical ICU survivors after implementation of a multidisciplinary clinic	Observation	70	54 (±15.7)	Trauma			Hospital discharge		18 ± 13.7
10	6MWT	Six-month and 12-month patient outcomes based on inflammatory subphenotypes in sepsis-associated ARDS: secondary analysis of SAILS-ALTOS	Observation	232	53 (40-65)	ARDS			Not mentioned	9 (7-14)	15 (10-22)
11	6MWT	One-Year Outcomes of Mechanically Ventilated COVID-19 ICU Survivors: A Prospective Cohort Study	Observation	94	63 (55–68)	COVID		15 (13–17)	Hospital discharge	20 (11–34)	32 (21–40)
12	6MWT	One-Year Mental and Physical Health Assessment in Survivors after Extracorporeal Membrane Oxygenation for COVID-19-related Acute Respiratory	Observation	62	47 (40–55)	ARDS			Hospital discharge	43 (33–62)	85 (29–112)
13	6MWT	Risk factors for physical impairment after acute lung injury in a national, multicenter study	Observation	203	48 (±15)	ALI			ICU admission	14 ± 11	
14	6MWT	Long-Term Survival and Health-Related Quality of Life in Adults After Extra Corporeal Membrane Oxygenation	Observation	33	42 (26.5–57)	ARDS	10 (6.5–12)	20 (16–26.5)	Hospital discharge	6 (3.2–17.5)	8.2 (3.6–17.1)
15	MoCA	Safety, feasibility and initial efficacy of an app-facilitated telerehabilitation (AFTER) programme for COVID-19 survivors: a pilot randomised study	Intervention	13	54 (±10)	COVID			Hospital discharge		8 ± 9
16	MoCA	Post-Intensive Care Syndrome in Non-COVID-19 ICU Survivors during the COVID-19 Pandemic in South Korea: A Multicenter Prospective Cohort Study	Observation	237	58.30 (±13.30)	Surgical	4.37 ± 2.61		Hospital discharge	4.02 ± 4.28)	
17	MoCA	Safety and Feasibility of an Interdisciplinary Treatment Approach to Optimize Recovery From Critical Coronavirus Disease 2019	Observation	32	55.5 (52–65)	ARDS	9.5 (4–11)	20 (12–24)	Hospital discharge	11 (9–18)	19.5 (13–28)
18	MoCA	ICU Recovery Clinic Attendance, Attrition, and Patient Outcomes: The Impact of Severity of Illness, Gender, and Rurality	Observation	38	53.2 (±16)	Sepsis<br>ARDS	9.4 ± 2.9		Hospital discharge	9.6 (7–16)	14 (11–22)
19	MoCA	Neuropsychiatric and Cognitive Outcomes in Patients 6 Months After COVID-19 Requiring Hospitalization Compared With Matched Control Patients Hospitalized for Non-COVID-19 Illness	Observation	85	56.8 (±14)	COVID			ICU admission	16 (9-26)	
20	IES-R	Factors associated with missed assessments in a 2-year longitudinal study of acute respiratory distress syndrome survivors	Observation	196	49 (40-58)	ARDS			Hospital discharge		
21	IES-R	Safety and Feasibility of an Interdisciplinary Treatment Approach to Optimize Recovery From Critical Coronavirus Disease 2019	Observation	32	55.5 (52–65)	ARDS	9.5 (4–11)	20 (12–24)	Hospital discharge	11 (9–18)	19.5 (13–28)
22	IES-R	One-Year Outcomes of Postintensive Care Syndrome in Critically Ill Coronavirus Disease 2019 Patients: A Single Institutional Study	Observation	18	57.5 (49.5–71.8)	COVID			ICU discharge	6(5.0–12.5)	23.5(18.0–39.5)
23	IES-R	Cognitive and psychosocial outcomes of mechanically ventilated intensive care patients with and without delirium	Observation	103	60 (±16)	Not mentioned			ICU discharge	3.0 (4.8-12.7)	
24	IES-R	Implementing an intensive care unit (ICU) diary program at a large academic medical center: Results from a randomized control trial evaluating psychological morbidity associated with critical illness	RCT	17	40 (31-51)	Abdominal aortic aneurysm rupture			ICU discharge	9 (5-24)	
25	IES-R	Virtual Reality to Improve Sequelae of the Postintensive Care Syndrome: A Multicenter, Randomized Controlled Feasibility Study	Intervention	25	59 (59–80)	Sepsis	2 (1-4)		Staring intervention	14 (3–81)	38 (17–116)
26	IES-R	Terminal weaning or immediate extubation for withdrawing mechanical ventilation in critically ill patients (the ARREVE observational study)	Observation	210	68 (±13)	Medical	8 ± 4		Death		
27	IES-R	Combination of delirium and coma predicts psychiatric symptoms at twelve months in critically ill patients: A longitudinal cohort study	Observation	81	68 (57–75)	Sepsis		21 (17–26)	ICU discharge	5 (3–9)	28 (15–42)

28	IES-R	Preventing Posttraumatic Stress in ICU Survivors: A Single-Center Pilot Randomized Controlled Trial of ICU Diaries and Psychoeducation	RCT	14	49.9 (±16.9)	Not mentioned	9.2 ± 3.5		ICU discharge	19.0 (9–24.5)	24.0 (20–47.5)
29	IES-R	ICU Recovery Clinic Attendance, Attrition, and Patient Outcomes: The Impact of Severity of Illness, Gender, and Rurality	Observation	38	53.2 (±16)	Sepsis<br>ARDS	9.4 ± 2.9		Hospital discharge	9.6 (7–16)	14 (11–22)
30	IES-R	Psychological distress and physical disability in patients sustaining severe injuries in road traffic crashes: Results from a one-year cohort study from three European countries	Observation	120	41.8 (±16.7)	Not mentioned			Hospital discharge		
31	IES-R	Timing of Exposure to ICU Diaries and Its Impact on Mental Health, Memories, and Quality of Life: A Double-Blind Randomized Control Trial	RCT	41	46.6 (±17.6)	Respiratory	5.9 (3.0)	13.7 (4.9)	Hospital discharge	8.2 ± 7.1	
32	IES-R	Psychologic Distress and Quality of Life After ICU Treatment for Coronavirus Disease 2019: A Multicenter, Observational Cohort Study	Observation	118	61 (36–77)	COVID			Hospital discharge	13 (0–49)	22 (1–67)
33	IES-R	Intensive Care Unit-Specific Virtual Reality for Critically Ill Patients With COVID-19: Multicenter Randomized Controlled Trial	RCT	44	59 (51-65)	COVID			Hospital discharge	14 (7-28)	24 (13-40)
34	IES-R	Activities of daily living status and psychiatric symptoms after discharge from an intensive care unit: a single-center 12-month longitudinal prospective study	Observation	117	71 (60–78)	Sepsis		21 (17–26)	Hospital discharge	5 (3–9)	28 (15–42)
35	IES-R	Six-month and 12-month patient outcomes based on inflammatory subphenotypes in sepsis-associated ARDS: secondary analysis of SAILS-ALTOS	Observation	232	53 (40-65)	ARDS			Not mentioned	9 (7-14)	15 (10-22)
36	IES-R	Influence on Depression, Anxiety, and Satisfaction of the Relatives' Visit to Intensive Care Units prior to Hospital Admission for Elective Cardiac Surgery: A Randomized Clinical Trial	RCT	19	52.42 (±16.56)	Not mentioned			ICU discharge	8 ± 12.95	
37	IES-R	Emotional disorders in pairs of patients and their family members during and after ICU stay	Observation	184	59.3 ±15.5 (18–92)	Not mentioned	2.38 (0–15)		ICU discharge	5.5 (2–47)	
38	HADS	Life after COVID-19: the road from intensive care back to living - a prospective cohort study	Observation	67	62 (57–68)	COVID			ICU discharge	20 (12-33)	
39	HADS	Factors associated with missed assessments in a 2-year longitudinal study of acute respiratory distress syndrome survivors	Observation	196	49 (40-58)	ARDS			Hospital discharge		
40	HADS	Safety and Feasibility of an Interdisciplinary Treatment Approach to Optimize Recovery From Critical Coronavirus Disease 2019	Observation	32	55.5 (52–65)	ARDS	9.5 (4–11)	20 (12–24)	Hospital discharge	11 (9–18)	19.5 (13–28)
41	HADS	One-Year Outcomes of Mechanically Ventilated COVID-19 ICU Survivors: A Prospective Cohort Study	Observation	94	63 (55–68)	COVID		15 (13–17)	Hospital discharge	20 (11–34)	32 (21–40)
42	HADS	Exercise-based rehabilitation after hospital discharge for survivors of critical illness with intensive care unit-acquired weakness: A pilot feasibility trial	Observation	10	68.5 (64.3-78.0)	Not mentioned	12.0 (7.5-14.3)	23.5 (21.0-30.3)	Hospital discharge	13.0 (9.8-20.5)	47.5 (26.5-68.5)
43	HADS	One-Year Outcomes of Postintensive Care Syndrome in Critically Ill Coronavirus Disease 2019 Patients: A Single Institutional Study	Observation	18	57.5 (49.5–71.8)	COVID			ICU discharge	6(5.0–12.5)	23.5(18.0–39.5)
44	HADS	Supervised exercise rehabilitation in survivors of critical illness: A randomised controlled trial	RCT	30	62.5 (46–70)	Surgical		13 (9–19)	Hospital discharge	7 (4–15)	15 (9–25)
45	HADS	Lack of clinically relevant correlation between subjective and objective cognitive function in ICU survivors: a prospective 12-month follow-up study	Observation	58	54 (41–64)	Cardiovascular		26 (22–30)	Hospital discharge	4.45 (2–8.5)	
46	HADS	Effects of a Telephone- and Web-based Coping Skills Training Program Compared with an Education Program for Survivors of Critical Illness and Their Family Members	RCT	86	49.7 (±13.8)	Not mentioned		25.4 ± 8.7	ICU discharge		
47	HADS	Mental health and quality of life outcomes in family members of patients with chronic critical illness admitted to the intensive care units of two Brazilian hospitals serving the extremes of the socioeconomic spectrum	Observation	100	69 (59.5–81.0)	Sepsis	6.0 (3.0–9.0)		Hospital discharge	16.0 (10.0–26.0)	38.0 (27.0–56.0)
48	HADS	Older patients' recovery following intensive care: A follow-up study with the RAIN questionnaire	Observation	82	73 (±6.39)	Not mentioned			ICU discharge	5 ±8.5	21 ±21.13
49	HADS	ICU Recovery Clinic Attendance, Attrition, and Patient Outcomes: The Impact of Severity of Illness, Gender, and Rurality	Observation	38	53.2 (±16)	Sepsis<br>ARDS	9.4 ± 2.9		Hospital discharge	9.6 (7–16)	14 (11–22)
50	HADS	Psychologic Distress and Quality of Life After ICU Treatment for Coronavirus Disease 2019: A Multicenter, Observational Cohort Study	Observation	118	61 (36–77)	COVID			Hospital discharge	13 (0–49)	22 (1–67)
51	HADS	Intensive Care Unit-Specific Virtual Reality for Critically Ill Patients With COVID-19: Multicenter Randomized Controlled Trial	RCT	44	59 (51-65)	COVID			Hospital discharge	14 (7-28)	24 (13-40)
52	HADS	Post-Intensive Care Syndrome in Non-COVID-19 ICU Survivors during the COVID-19 Pandemic in South Korea: A Multicenter Prospective Cohort Study	Observation	237	58.30 (±13.30)	Surgical	4.37 ± 2.61		Hospital discharge	4.02 ± 4.28	
53	HADS	Activities of daily living status and psychiatric symptoms after discharge from an intensive care unit: a single-center 12-month longitudinal prospective study	Observation	117	71 (60–78)	Sepsis		21 (17–26)	Hospital discharge	5 (3–9)	28 (15–42)
54	HADS	Six-month and 12-month patient outcomes based on inflammatory subphenotypes in sepsis-associated ARDS: secondary analysis of SAILS-ALTOS	Observation	232	53 (40-65)	ARDS			Not mentioned	9 (7-14)	15 (10-22)

55	HADS	Influence on Depression, Anxiety, and Satisfaction of the Relatives' Visit to Intensive Care Units prior to Hospital Admission for Elective Cardiac Surgery: A Randomized Clinical Trial	RCT	19	52.42 (±16.56)	Not mentioned			ICU discharge	8 ± 12.95	
56	HADS	Emotional distress in neuro-ICU survivor-caregiver dyads: The recovering together randomized clinical trial	RCT	58	Not mentioned	Neurology			Staring intervention		
57	HADS	Impact of Clinical and Quality of Life Outcomes of Long-Stay ICU Survivors Recovering From Rehabilitation on Caregivers' Burden	Observation	23	68 (57–74)	Surgical			Hospital discharge	25 (21–39)	74 (31–95)
58	HADS	Health-related quality of life, anxiety and depression and physical recovery after critical illness - A prospective cohort study	Observation	79	67 (59–74)	Not mentioned		22 (17–27)	Hospital discharge	4 (2–7)	19 (11–33)
59	HADS	Emotional disorders in pairs of patients and their family members during and after ICU stay	Observation	184	59.3 ±15.5 (18–92)	Not mentioned	2.38 (0–15)		ICU discharge	5.5 (2–47)	
60	HADS	Prevalence and Risk Factor Analysis of Post-Intensive Care Syndrome in Patients with COVID-19 Requiring Mechanical Ventilation: A Multicenter Prospective Observational Study	Observation	147	68 (60-75)	COVID	5 (4-7)		ICU discharge	13 (8-21)	26 (15-51)
61	HADS	Intensive care survivor-reported symptoms: a longitudinal study of survivors' symptoms	Observation	118	55·1 (±14·4)	Not mentioned			ICU discharge	9 (5–15)	
62	HADS	The Impact of Resilience Factors and Anxiety During Hospital Admission on Longitudinal Anxiety Among Dyads of Neurocritical Care Patients Without Major Cognitive Impairment and Their Family Caregivers	Observation	102	52 (±17)	Cerebrovascular			ICU admission		
63	HADS	Early psychological screening of intensive care unit survivors: a prospective cohort study	Observation	82	62 (41–70)	Medical		10 (7–14)	ICU discharge	3 (2–6)	
64	HADS	Six-Month Outcomes in COVID-19 ICU Patients and Their Family Members: A Prospective Cohort Study	Observation	60	62.5 (55.3–68.0)	COVID			ICU discharge	19.4 (12.3–31.7)	30.6 (21.9–44.7)
65	HADS	Effect of supervised aerobic exercise rehabilitation on physical fitness and quality-of-life in survivors of critical illness: an exploratory minimized controlled trial (PIX study)	Intervention	30	40.5 (19–60)	Sepsis<br>Trauma		16.4 ± 7.8	Hospital discharge		
66	HADS	Reprint of Recovery programme for ICU survivors has no effect on relatives' quality of life: Secondary analysis of the RAPIT-study	RCT	94	61.0 (41.75–69.0)	Sepsis		26 (20.5–32.5)	ICU discharge	12 (5–21.25)	
67	HADS	Effect of a condolence letter on grief symptoms among relatives of patients who died in the ICU: a randomized clinical trial	RCT	119	61 (54–66)	Not mentioned			Death		
68	HADS	Baseline resilience and depression symptoms predict trajectory of depression in dyads of patients and their informal caregivers following discharge from the Neuro-ICU	Observation	102	52 (±17)	Cerebrovascular			Hospital discharge		
69	SF-36	Factors associated with missed assessments in a 2-year longitudinal study of acute respiratory distress syndrome survivors	Observation	196	49 (40-58)	ARDS			Hospital discharge		
70	SF-36	Association Between Tracheostomy and Functional, Neuropsychological, and Healthcare Utilization Outcomes in the RECOVER Cohort	Observation	224	58.6 (±15.4)	Not mentioned		22.5 ± 7.8	ICU discharge	16 (12-22)	31 (22-46)
71	SF-36	Exercise-based rehabilitation after hospital discharge for survivors of critical illness with intensive care unit-acquired weakness: A pilot feasibility trial	Observation	10	68.5 (64.3-78.0)	Not mentioned	12.0 (7.5-14.3)	23.5 (21.0-30.3)	Hospital discharge	13.0 (9.8-20.5)	47.5 (26.5-68.5)
72	SF-36	Intensive Care Unit-Specific Virtual Reality for Critically Ill Patients With COVID-19: Multicenter Randomized Controlled Trial	RCT	44	59 (51-65)	COVID			Hospital discharge	14 (7-28)	24 (13-40)
73	SF-36	Physical, cognitive and mental health outcomes in 1-year survivors of COVID-19-associated ARDS	Observation	98	Not mentioned	ARDS			ICU discharge		
74	SF-36	Long-term recovery following critical illness in an Australian cohort	Observation	56	59 (±14.1)	Cardiovascular		20 ± 7	ICU discharge	7 (6–11)	22 (15–36)
75	SF-36	Health-related quality of life in ICU survivors-10 years later	Observation	149	Not mentioned	Not mentioned		19 (14–23)	ICU discharge	8 (5–15)	23 (13–39.5)
76	SF-36	Longitudinal Assessment of Health and Quality of Life of COVID-19 Patients Requiring Intensive Care-An Observational Study	Observation	18	54 (±12.3)	COVID	3.5 (1–16)	15.5 ± 8.3	Hospital discharge	10 (1–71)	21.5 (8–71)
77	SF-36	Comorbidities Might Condition the Recovery of Quality of Life in Survivors of	Observation	79	53.9 (±22.6)	Sepsis		19 ± 3.5	ICU discharge	8.1 ± 9.3	28.5 ± 31.1
78	SF-36	Physical complications in acute lung injury survivors: a two-year longitudinal prospective study	Observation	222	49 (40-58)	Pneumonia	11 (8-15)	26 (20-33)	ICU discharge	13 (7-21)	
79	SF-36	ICU survivors show no decline in health-related quality of life after 5 years	Observation	749	71 (62–77)	Not mentioned		19 (14–23)	ICU discharge	8 (5–15)	23 (13–39.5)
80	SF-36	Long-Term Outcome after Prolonged Mechanical Ventilation	Observation	132	64 (56–71)	Surgical		14 (10–16)	Hospital discharge		
81	SF-36	One-year outcome of patients admitted after cardiac arrest compared to other causes of ICU admission	Observation	114	61 (50-72)	Cardiac arest			ICU discharge	12 (7-21)	
82	SF-36	Quality of life of critically ill patients in a developing country: a prospective longitudinal study	Observation	75	50.2 (±17.1)	Surgical		14.0 (11.0–17.0)	Hospital discharge	13.0 (7.0–17.0)	29.0 (20.0–48.0)
83	SF-36	A Study of ICU Outcome and Long-Term Quality of Life in ICU Survivors in Central India	Observation	82	47.81 (±17.29)	Not mentioned	6.91 ± 3.81	12.08 ± 4.40	Hospital discharge	6.73 ± 4.10	
84	SF-36	Long-term quality of life in critically ill patients with acute kidney injury treated with renal replacement therapy: a matched cohort study	Observation	47	57 (45–69)	AKI	9 (5–11)	26 (21–31)	ICU discharge	22 (11–42)	70 (30–100)

85	SF-36	Critically ill octogenarians and nonagenarians: evaluation of long-term outcomes, posthospital trajectories and quality of life one year and seven years after ICU discharge	Observation	93	83 (81-85)	Not mentioned	4 (3-8)	20 (15-24)	ICU discharge	3 (2-5)	17 (9-38)
86	SF-36	The fear and risk of community falls in patients following an intensive care admission: An exploratory cohort study	Observation	12	54.5 (44.5-59.0)	Not mentioned			ICU discharge	9.0 (7.2-14.5)	
87	SF-36	Long-Term Disabilities of Survivors of Out-of-Hospital Cardiac Arrest: The Hanox Study	Observation	74	56 (47-66)	Cardiac arest	10 (8-12)		ICU admission	6 (4-11)	
88	SF-36	Implications for post critical illness trial design: sub-phenotyping trajectories of functional recovery among sepsis survivors	Observation	159	Not mentioned	Sepsis			ICU admission	23.0 (12.8–39.5)	
89	SF-36	Neurocognitive and Quality-of-life Outcomes Following Intensive Care Admission: A Prospective 6-month Follow-up Study	Observation	136	56 (49.2–65.7)	COPD<br>ARDS	12 (10–14)	25.5 (22–28)	ICU discharge	12 (8–14)	
90	SF-36	Effect of supervised aerobic exercise rehabilitation on physical fitness and quality-of-life in survivors of critical illness: an exploratory minimized controlled trial (PIX study)	Intervention	30	40.5 (19–60)	Sepsis<br>Trauma		16.4 ± 7.8	Hospital discharge		
91	SF-36	Reprint of Recovery programme for ICU survivors has no effect on relatives' quality of life: Secondary analysis of the RAPIT-study	RCT	94	61.0 (41.75–69.0)	Sepsis		26 (20.5–32.5)	ICU discharge	12 (5–21.25)	
92	SF-36	Health-related quality of life, anxiety and depression and physical recovery after critical illness - A prospective cohort study	Observation	79	67 (59–74)	Not mentioned		22 (17–27)	Hospital discharge	4 (2–7)	19 (11–33)
93	SF-36	Six-month and 12-month patient outcomes based on inflammatory subphenotypes in sepsis-associated ARDS: secondary analysis of SAILS-ALTOS	Observation	232	53 (40-65)	ARDS			Not mentioned	9 (7-14)	15 (10,22)
94	SF-36	Quality of Life and 1-Year Survival in Patients With Early Septic Shock: Long-Term Follow-Up of the Australasian Resuscitation in Sepsis Evaluation Trial	Intervention	798	63.1 ± 16.5	Sepsis		15.8 ± 6.5	Staring study		
95	SF-36	Feasibility of a home-based interdisciplinary rehabilitation program for patients with Post-Intensive Care Syndrome: the REACH study	Intervention	24	54 (±23)	Respiratory			Hospital discharge	11 ± 12	34.5 ± 28
96	SF-36	Long-Term Survival and Health-Related Quality of Life in Adults After Extra Corporeal Membrane Oxygenation	Observation	33	42 (26.5–57)	ARDS	10 (6.5–12)	20 (16–26.5)	Hospital discharge	6 (3.2–17.5)	8.2 (3.6–17.1)
97	SF-36	Health-Related Quality of Life in Australasian Survivors of H1N1 Influenza Undergoing Mechanical Ventilation	Observation	722	40 (26–54)	Influenza		18.0 (14–20)	ICU discharge	12.5 (7–27)	20.0 (15–38)
98	PICS-F	Coping strategies, anxiety and depressive symptoms in family members of patients treated with extracorporeal membrane oxygenation: A prospective cohort	Observation	26	47 (±18)	(Family member)			Staring intervention	17 ± 26.50	30,23 ± 64.72
99	PICS-F	Emotional distress in neuro-ICU survivor-caregiver dyads: The recovering together randomized clinical trial	RCT	58	Not mentioned	(Family member)			Staring intervention		
100	PICS-F	Six-Month Outcomes in COVID-19 ICU Patients and Their Family Members: A Prospective Cohort Study	Observation	78	56.0 (41.0–63.0)	(Family member)			ICU discharge	19.4 (12.3–31.7)	30.6 (21.9–44.7)
101	PICS-F	Emotional Experiences and Coping Strategies of Family Members of Critically Ill Patients	Observation	40	52 (±12)	(Family member)			ICU discharge		
102	PICS-F	Post-intensive care syndrome following cardiothoracic critical care: Feasibility of a complex intervention	Observation	27	66 (61–75)	(Family member)			Hospital discharge	13 (9–21)	
103	PICS-F	Baseline resilience and depression symptoms predict trajectory of depression in dyads of patients and their informal caregivers following discharge from the Neuro-ICU	Observation	103	53 (±14)	(Family member)			Hospital discharge		
104	PICS-F	Feasibility and Efficacy of a Resiliency Intervention for the Prevention of Chronic Emotional Distress Among Survivor-Caregiver Dyads Admitted to the Neuroscience Intensive Care Unit: A Randomized Clinical Trial	RCT	29	50.1 (±16.4)	(Family member)			ICU admission		
105	PICS-F	Post-intensive care syndrome symptoms and health-related quality of life in family decision-makers of critically ill patients	Observation	48	62.5 (22–90)	(Family member)			ICU admission		
